# Genome Sequence of *Borrelia garinii* Strain SZ, Isolated in China

**DOI:** 10.1128/genomeA.00010-14

**Published:** 2014-07-17

**Authors:** Qiong Wu, Zhijie Liu, Youquan Li, Guiquan Guan, Qingli Niu, Ze Chen, Jianxun Luo, Hong Yin

**Affiliations:** State Key Laboratory of Veterinary Etiological Biology, Key Laboratory of Veterinary Parasitology of Gansu Province, Lanzhou Veterinary Research Institute, Chinese Academy of Agricultural Sciences, Lanzhou, Gansu, People’s Republic of China

## Abstract

We announce the genome sequence of *Borrelia garinii* strain SZ, isolated from *Dermacentor* ticks collected in northeastern China. *B. garinii* strain SZ carries numerous plasmids, both 10 circular and 9 linear plasmids. The 902,487-bp linear chromosome (28.2% GC content) contains 820 open reading frames, 33 tRNAs, and 4 complete rRNAs. The plasmid cp32-10 contains one clustered regularly interspaced short palindromic repeat (CRISPR) with four repeats.

## GENOME ANNOUNCEMENT

*Borrelia burgdorferi* is the agent of Lyme disease, which is caused by infection with the tick-borne spirochete *Borrelia burgdorferi sensu lato* complex. The species complex currently consists of 20 proposed and confirmed species (http://www.ncbi.nlm.nih.gov/Taxonomy), of which *B. burgdorferi sensu stricto*, *B. afzelii*, *B. garinii*, *B. lusitaniae, B. spielmanii*, *B. valaisiana*, and *B. bissettii* are associated with human Lyme disease (1–3). During recent years, there has been an extraordinary accumulation of knowledge on the genomics of these organisms. To date, whole-genome sequences have been reported for 26 *B. burgdorferi* isolates, including 14 *B. burgdorferi sensu stricto*, 3 *B. afzelii*, and 5 *B. garinii* isolates and 1 *B. bissettii*, 1 *B. valaisiana*, 1 *B. spielmanii*, and 1 *B. bavariensis* sp. nov. isolate (4–9). Since the epidemiological investigation of Lyme disease in 1986, more than 100 *Borrelia* strains have been isolated from 30 provinces of China (10, 11). The whole-genome sequences of *B. afzelii* HLJ01 and *B. garinii* NMJW1 have been reported (7, 8). The whole-genome sequences of other Chinese isolates have not yet been reported.

To further understanding of the genomic information and genetic polymorphisms of *B. burgdorferi* isolates in China, we announce here the whole-genome sequence of *B. garinii* SZ, which was isolated from *Dermacentor* ticks collected in the Heilongjiang Province of China (12) and triggers multisystem pathological damage in mice (13). DNA from a low-passage-number culture was sequenced to minimize plasmid loss, and sequencing proceeded to about 8-fold coverage by use of Illumina HiSeq 2000 technology. Solexa sequencing technology was used to close gaps and exclude scaffolded regions in the sequences, and then CLC Workbench 6.0 was used to *de novo* assemble the 7,068,070 paired-end Illumina sequencing reads. Based on this assembly, the interscaffold and intrascaffold gaps were closed by local assembly. Gene prediction was performed using Glimmer3.02. tRNAScan-SE1.23 was used to search for tRNA genes and RNAmmer1.2 to search for rRNA genes. Protein BLAST was run, using the translated coding sequences as a query against the reference sequence. Clustered regularly interspaced short palindromic repeat (CRISPR) analysis was run by CRT1.2.

Like other *Borrelia* species, this isolate was found to carry numerous plasmids, both linear and circular. The linear *B. garinii* SZ chromosome includes 902,487 bp in total (28.2% GC content) and carries 33 tRNAs and 4 complete rRNAs. On the chromosome are 820 open reading frames (ORFs), of which 35% code for hypothetical proteins. This strain is highly similar to *B. garinii* NMJW1 and *B. garinii* Bgvir. Plasmids cp26, cp32, lp17, lp28, lp36, lp38, lp54, and lp56 are universally present in *B. garinii* SZ, as they are in *B. burgdorferi* isolates, and the overall gene contents of these plasmids are rather similar to those of the plasmids of *B. burgdorferi*. However, *B. garinii* SZ contains more cp32 plasmids than other *B. garinii* isolates; their gene contents vary considerably. The plasmid cp32-10 contains one CRISPR with four repeats (our unpublished data). In-depth comparative analysis among different species is now the focus of our work.

This genome sequence contributes to a solid foundation for understanding *B. burgdorferi sensu lato* diversity and providing clues for the pathogenesis of Lyme disease.

### Nucleotide sequence accession number.

The *B. garinii* strain SZ genome sequence has been deposited in the NCBI database with the accession number CP007564.

## References

[1] Piesman J Gern L 2004 Lyme borreliosis in Europe and North America. Parasitology 129(Suppl):191–220. 10.1017/S0031182004005402 15938512

[2] Scholz HC Margos G Derschum H Speck S Tserennorov D Erdenebat N Undraa B Enkhtuja M Battsetseg J Otgonchimeg C 2012 High prevalence of genetically diverse *Borrelia bavariensis*-like strains in *Ixodes persulcatus* from Selenge Aimag, Mongolia. Ticks Tick Borne Dis. 4:89–92. 10.1016/j.ttbdis.2012.08.004 23084366

[3] Rudenko N Golovchenko M Růzõek D Piskunova N Mallátová N Grubhoffer L 2009 Molecular detection of *Borrelia bissettii* DNA in serum samples from patients in the Czech Republic with suspected borreliosis. FEMS Microbiol. Lett. 292:274–281. 10.1111/j.1574-6968.2009.01498.x 19187198

[4] Schutzer SE Fraser-Liggett CM Casjens SR Qiu WG Dunn JJ Mongodin EF Luft BJ 2011 Whole-genome sequences of thirteen isolates of *Borrelia burgdorferi*. J. Bacteriol. 193:1018–1020. 10.1128/JB.01158-10 20935092 PMC3028687

[5] Casjens SR Mongodin EF Qiu WG Dunn JJ Luft BJ Fraser-Liggett CM Schutzer SE 2011 Whole-genome sequences of two *Borrelia afzelii* and two *Borrelia garinii* Lyme disease agent isolates. J. Bacteriol. 193:6995–6996. 10.1128/JB.05951-11 22123755 PMC3232841

[6] Casjens SR Fraser-Liggett CM Mongodin EF Qiu WG Dunn JJ Luft BJ Schutzer SE 2011 Whole-genome sequence of an unusual *Borrelia burgdorferi* sensu lato isolate. J. Bacteriol. 193:1489–1490. 10.1128/JB.01521-10 21217002 PMC3067611

[7] Jiang B Yao H Tong Y Yang X Huang Y Jiang J Cao W 2012 Genome sequence of *Borrelia garinii* strain NMJW1, isolated from China. J. Bacteriol. 194:6660–6661. 10.1128/JB.01844-12 23144406 PMC3497544

[8] Jiang BG Zheng YC Tong YG Jia N Huo QB Fan H Ni XB Ma L Yang XF Jiang JF Cao WC 2012 Genome sequence of *Borrelia afzelii* strain HLJ01, isolated from a patient in China. J. Bacteriol. 194:7014–7015. 10.1128/JB.01863-12 23209254 PMC3510575

[9] Fraser CM Casjens S Huang WM Sutton GG Clayton R Lathigra R White O Ketchum KA Dodson R Hickey EK Gwinn M Dougherty B Tomb JF Fleischmann RD Richardson D Peterson J Kerlavage AR Quackenbush J Salzberg S Hanson M van Vugt R Palmer N Adams MD Gocayne J Weidman J Utterback T Watthey L McDonald L Artiach P Bowman C Garland S Fuji C Cotton MD Horst K Roberts K Hatch B Smith HO Venter JC 1997 Genomic sequence of a Lyme disease spirochaete, *Borrelia burgdorferi*. Nature 390:580–586. 10.1038/37551 9403685

[10] Zhang ZF Wan KL Zhang JS Chu K Dou G Li M Chan L Liang C Hou T Wong H 1997 Studies on epidemiology and etiology of Lyme disease in China. Zhonghua Liu Xing Bing Xue Za Zhi 18:8–11 (In Chinese.) 9812472

[11] Hao Q Hou X Geng Z Wan K 2011 Distribution of *Borrelia burgdorferi* sensu lato in China. J. Clin. Microbiol. 49:647–650. 10.1128/JCM.00725-10 21106783 PMC3043522

[12] Niu Q Yang J Guan G Fu Y Ma M Li Y Liu J Liu Z Liu A Ren Q 2010 Identification and phylogenetic analysis of Lyme disease *Borrelia* spp. isolated from Shangzhi Prefecture of Heilongjiang Province. Chin. Vet. Sci. 40:551–556

[13] Wu Q Liu Z Wang J Li Y Guan G Yang J Chen Z Luo J Yin H 2013 Pathogenic analysis of *Borrelia garinii* strain SZ isolated from northeastern China. Parasites Vectors 6:177–177. 10.1186/1756-3305-6-177 23773815 PMC3689080

